# Supplementary material for: Amyloid burden quantification depends on PET and MR image processing methodology
Source: PLoS One. 2021 Mar 5;16(3):e0248122. doi: 10.1371/journal.pone.0248122 (PMC7935288; doi:10.1371/journal.pone.0248122)
Supplement: S1 File — Include tables with additional information about parameters for spatial transformations, descriptive statistics of the metrics presented in the manuscript, and a table of abbreviations. (DOCX) [file pone.0248122.s001.docx]

**Table A:** SPM spatial normalisation parameters for MAPa and MAPc

| Parameter | MAPa | MAPc | USM |
| --- | --- | --- | --- |
| Template image | T1 MRI | T1 MRI | N/A |
| Template Weighting Image | N/A | N/A | N/A |
| Source image smoothing [mm] | 8 | 8 | N/A |
| Template image smoothing [mm] | 0 | 0 | N/A |
| Affine regularisation | ICBM space template | ICBM space template | ICBM space template (European brains) |
| Nonlinear frequency cut-off | 25 | 25 | N/A |
| Nonlinear iterations | 16 | 16 | N/A |
| Nonlinear regularisation | 1 | 1 | N/A |
| Preserve | Total amount | Concentrations | N/A |
| Bounding box | -90 -126 -72  90 90 108 | -90 -126 -72  90 90 108 | -90 -126 -72  90 90 108 |
| Voxel size | 2 2 2 | 2 2 2 | 2 2 2 |
| Interpolation | Trilinear | Trilinear | Trilinear |
| Bias regularisation | N/A | N/A | Very light (0.0001) |
| Bias full width at half maximum (FWHM) | N/A | N/A | 60 mm cut-off |
| Tissue probability map | N/A | N/A | 6 maps in MNI space |
| Wrapping Regularisation | N/A | N/A | 0 0.001 0.5 0.05 0.2 |
| Smoothness | N/A | N/A | 0 |
| Sampling distance | N/A | N/A | 3 |

SPM parameters for spatial normalisation methods. USM is the default SPM12 spatial normalisation method, while the MAP methods available through the SPM > Tools > Old Normalise menu in the Batch Editor on SPM12 (MAPc is the default “Preserve” option).

**Table B:** Descriptive statistics of intensity metrics

| Space | Spatial Normalisation Method | Grey Matter Segmentation | SUVR_mean_ | | | | SUVR_meanAβ+_ | | | |
| --- | --- | --- | --- | --- | --- | --- | --- | --- | --- | --- |
|  |  |  | HC | | AD | | HC | | AD | |
|  |  |  | Mean | SD | Mean | SD | Mean | SD | Mean | SD |
| Native Space | MAPa^†^ | MRn | 1.02 | 0.07 | 1.32 | 0.16 | 1.67 | 0.04 | 1.80 | 0.10 |
| Native Space | MAPa | TEs | 1.00 | 0.07 | 1.25 | 0.19 | 1.71 | 0.03 | 1.79 | 0.09 |
| Native Space | MAPc^†^ | MRn | 1.02 | 0.07 | 1.32 | 0.16 | 1.67 | 0.04 | 1.80 | 0.10 |
| Native Space | MAPc | TEs | 1.00 | 0.07 | 1.25 | 0.19 | 1.71 | 0.03 | 1.79 | 0.09 |
| Native Space | USM^†^ | MRn | 1.06 | 0.07 | 1.59 | 0.25 | 1.68 | 0.04 | 1.98 | 0.20 |
| Native Space | USM | TEs | 0.97 | 0.06 | 1.46 | 0.25 | 1.70 | 0.03 | 1.97 | 0.18 |
| Standard Space | MAPa | MRn | 1.01 | 0.09 | 1.32 | 0.16 | 1.67 | 0.04 | 1.78 | 0.08 |
| Standard Space | MAPa | MRs | 1.00 | 0.09 | 1.32 | 0.16 | 1.66 | 0.04 | 1.78 | 0.08 |
| Standard Space | MAPa | TEs | 0.99 | 0.07 | 1.24 | 0.20 | 1.70 | 0.03 | 1.78 | 0.06 |
| Standard Space | MAPc | MRn | 1.01 | 0.07 | 1.33 | 0.16 | 1.66 | 0.04 | 1.79 | 0.10 |
| Standard Space | MAPc | MRs | 1.02 | 0.07 | 1.33 | 0.16 | 1.65 | 0.04 | 1.79 | 0.10 |
| Standard Space | MAPc | TEs | 0.99 | 0.06 | 1.25 | 0.19 | 1.70 | 0.03 | 1.79 | 0.09 |
| Standard Space | USM | MRn | 1.06 | 0.07 | 1.60 | 0.25 | 1.67 | 0.04 | 1.99 | 0.21 |
| Standard Space | USM | MRs | 1.06 | 0.07 | 1.60 | 0.25 | 1.67 | 0.04 | 1.99 | 0.21 |
| Standard Space | USM | TEs | 0.98 | 0.06 | 1.47 | 0.25 | 1.70 | 0.03 | 1.97 | 0.18 |

Descriptive statistics of intensity metrics derived from all image processing pipelines.

^†^ Spatial normalization only refers to the inverse transformation of the cerebellum VOI from Standard Space to Native Space

**Table C:** Descriptive statistics of volumetric metrics

| Space | Spatial Normalisation Method | Grey Matter Segmentation | Aβ+ volume | | | | Amyloid Fractional Volume | | | |
| --- | --- | --- | --- | --- | --- | --- | --- | --- | --- | --- |
|  |  |  | HC | | AD | | HC | | AD | |
|  |  |  | Mean | SD | Mean | SD | Mean | SD | Mean | SD |
| Native Space | MAPa^†^ | MRn | 47.6 | 30.4 | 206 | 94 | 7.4% | 4.7% | 35.3% | 14.7% |
| Native Space | MAPa | TEs | 74.3 | 35.0 | 275 | 129 | 8.9% | 4.1% | 31.6% | 13.2% |
| Native Space | MAPc^†^ | MRn | 47.6 | 30.4 | 206 | 94 | 7.4% | 4.7% | 35.3% | 14.7% |
| Native Space | MAPc | TEs | 74.3 | 35.0 | 275 | 129 | 8.9% | 4.1% | 31.6% | 13.2% |
| Native Space | USM^†^ | MRn | 62.2 | 34.2 | 308 | 111 | 9.6% | 5.4% | 53.3% | 17.4% |
| Native Space | USM | TEs | 69.9 | 36.0 | 410 | 161 | 7.8% | 3.9% | 45.4% | 16.2% |
| Standard Space | MAPa | MRn | 46.4 | 37.6 | 228 | 108 | 6.7% | 5.3% | 35.2% | 16.3% |
| Standard Space | MAPa | MRs | 54.4 | 45.5 | 254 | 118 | 6.4% | 5.2% | 34.6% | 16.2% |
| Standard Space | MAPa | TEs | 88.1 | 45.5 | 331 | 154 | 8.2% | 4.2% | 30.8% | 14.3% |
| Standard Space | MAPc | MRn | 54.6 | 36.6 | 255 | 107 | 6.6% | 4.6% | 35.8% | 15.1% |
| Standard Space | MAPc | MRs | 56.6 | 39.1 | 269 | 116 | 6.5% | 4.6% | 35.7% | 15.2% |
| Standard Space | MAPc | TEs | 87.8 | 42.0 | 337 | 145 | 8.2% | 3.9% | 31.4% | 13.5% |
| Standard Space | USM | MRn | 74.5 | 40.1 | 376 | 120 | 9.6% | 5.5% | 54.5% | 17.5% |
| Standard Space | USM | MRs | 74.2 | 42.5 | 383 | 125 | 9.2% | 5.4% | 53.8% | 17.1% |
| Standard Space | USM | TEs | 82.0 | 41.8 | 496 | 174 | 7.6% | 3.9% | 46.1% | 16.2% |

Descriptive statistics of volumetric metrics derived from all image processing pipelines.

^†^ Spatial normalization only refers to the inverse transformation of the cerebellum VOI from Standard Space to Native Space

**Table D:** Descriptive statistics of Total Amyloid Burden (TAB)

| Space | Spatial Normalisation Method | Grey Matter Segmentation | Total Amyloid Burden | | | |
| --- | --- | --- | --- | --- | --- | --- |
|  |  |  | HC | | AD | |
|  |  |  | Mean | SD | Mean | SD |
| Native Space | MAPa^†^ | MRn | 80 | 54 | 377 | 189 |
| Native Space | MAPa | TEs | 128 | 63 | 499 | 260 |
| Native Space | MAPc^†^ | MRn | 80 | 54 | 377 | 189 |
| Native Space | MAPc | TEs | 128 | 63 | 499 | 260 |
| Native Space | USM^†^ | MRn | 106 | 62 | 627 | 265 |
| Native Space | USM | TEs | 120 | 65 | 827 | 381 |
| Standard Space | MAPa | MRn | 79 | 66 | 411 | 204 |
| Standard Space | MAPa | MRs | 92 | 80 | 459 | 225 |
| Standard Space | MAPa | TEs | 151 | 81 | 595 | 289 |
| Standard Space | MAPc | MRn | 92 | 65 | 464 | 218 |
| Standard Space | MAPc | MRs | 95 | 69 | 490 | 237 |
| Standard Space | MAPc | TEs | 150 | 75 | 610 | 296 |
| Standard Space | USM | MRn | 126 | 73 | 762 | 287 |
| Standard Space | USM | MRs | 126 | 77 | 777 | 296 |
| Standard Space | USM | TEs | 140 | 76 | 997 | 412 |

Descriptive statistics of Total Amyloid Burden derived from all image processing pipelines.

^†^ Spatial normalization only refers to the inverse transformation of the cerebellum VOI from Standard Space to Native Space

**Table E:** List of abbreviations

| AD | Alzheimer’s Disease |
| --- | --- |
| AFV | Amyloid fractional volume |
| Aβ | β-amyloid |
| Aβ+ | [^11^C]PiB positive voxels |
| BMI | Body Mass Index |
| CER | Cerebellum |
| CERs | Cerebellum VOI originally available in Standard Space |
| CT | Computed tomography |
| GEE | Generalised estimating equations |
| GM | Grey matter |
| HC | Healthy volunteers |
| IQR | Interquartile range |
| MAP | Maximum a posteriori |
| MAPa | MAP while preserving total signal amount in the image |
| MAPc | MAP while preserving voxel concentrations in the image |
| MMSE | Mini-Mental State Examination |
| MNI | Montreal Neurological Institute |
| MRI | Magnetic resonance imaging |
| MRn | Tissue segmentation from MRI in Native Space |
| MRs | Tissue segmentation from MRI in Standard Space |
| NIA-AA | National Institute on Aging and Alzheimer’s Association |
| PET | Positron Emission Tomography |
| SPM | Statistical Parametric Mapping |
| SUVR | Standardized uptake value ratio |
| SUVR_mean_ | Average SUVR within GM VOI |
| SUVR_meanAβ+_ | Average SUVR within GM VOI for Aβ+ voxels |
| TAB | Total amyloid burden |
| TEs | Grey matter template originally available in Standard Space |
| TPM | Tissue probability maps |
| UMCG | University Medical Center Groningen |
| USM | Unified segmentation method |
| VOI | Volumes of interest |
